# Serum Potassium Levels and Its Variability in Incident Peritoneal Dialysis Patients: Associations with Mortality

**DOI:** 10.1371/journal.pone.0086750

**Published:** 2014-01-27

**Authors:** Qingdong Xu, Fenghua Xu, Li Fan, Liping Xiong, Huiyan Li, Shirong Cao, Xiaoyan Lin, Zhihua Zheng, Xueqing Yu, Haiping Mao

**Affiliations:** Department of Nephrology, The First Affiliated Hospital, Sun Yat-sen University, Key Laboratory of Nephrology, Ministry of Health of China, Guangzhou, China; University of Sao Paulo Medical School, Brazil

## Abstract

**Background:**

Abnormal serum potassium is associated with an increased risk of mortality in dialysis patients. However, the impacts of serum potassium levels on short- and long-term mortality and association of potassium variability with death in peritoneal dialysis (PD) patients are uncertain.

**Methods:**

We examined mortality-predictability of serum potassium at baseline and its variability in PD patients treated in our center January 2006 through December 2010 with follow-up through December 2012. The hazard ratios (HRs) were used to assess the relationship between baseline potassium levels and short-term (≤1 year) as well as long-term (>1 year) survival. Variability of serum potassium was defined as the coefficient of variation of serum potassium (CVSP) during the first year of PD.

**Results:**

A total of 886 incident PD patients were enrolled, with 248 patients (27.9%) presented hypokalemia (serum potassium <3.5 mEq/L). During a median follow-up of 31 months (range: 0.5–81.0 months), adjusted all-cause mortality hazard ratio (HR) and 95% confidence interval (CI) for baseline serum potassium of <3.0, 3.0 to <3.5, 3.5 to <4.0, 4.5 to <5.0, and ≥5.0 mEq/L, compared with 4.0 to <4.5 (reference), were 1.79 (1.02–3.14), 1.15 (0.72–1.86), 1.31 (0.82–2.08), 1.33 (0.71–2.48), 1.28 (0.53–3.10), respectively. The increased risk of lower potassium with mortality was evident during the first year of follow-up, but vanished thereafter. Adjusted all-cause mortality HR for CVSP increments of 7.5% to <12.0%; 12.0% to <16.7% and ≥16.7%, compared with <7.5% (reference), were 1.35 (0.67–2.71), 2.00 (1.05–3.83) and 2.18 (1.18–4.05), respectively. Similar association was found between serum potassium levels and its variability and cardiovascular mortality.

**Conclusions:**

A lower serum potassium level was associated with all-cause and cardiovascular mortality during the first year of follow-up in incident PD patients. In addition, higher variability of serum potassium levels conferred an increased risk of death in this population.

## Introduction

Disorder of potassium homeostasis may contribute to a higher risk of death in patients on dialysis. In patients with chronic kidney disease or those under hemodialysis (HD), a link between serum potassium levels and mortality is evident, with mortality risk significantly greater at potassium <4.0 mEq/L [1], [2]. Unlike HD patients, hypokalemia (serum potassium <3.5 mEq/L) is common in peritoneal dialysis (PD) patients, at a frequency ranging from 10 to 36% [3], [4], [5]. The reasons for this wide range of prevalence of hypokalemia are unknown, but may depend on studying different populations, time point of baseline serum potassium levels, and study period.

It has been well documented that low potassium levels are associated with general and sudden death among patients with cardiovascular disease [6], [7]. Among Chinese continuous ambulatory peritoneal dialysis (CAPD) patients, Szeto *et al.* has demonstrated that hypokalemia at baseline is an independent prognostic indicator of survival [8]. Recently, a large study from the United States showed that a time-averaged, but not baseline, serum potassium <3.5 mEq/L was associated with a higher adjusted risk for all-cause and cardiovascular mortality in a cohorts of prevalent PD patients [9]. Low potassium may affect myocardial resting membrane potential, repolarization and conduction velocity, suggesting that it causes negative short-term effects on mortality and becomes weaker following correction over longer period of time. However, the time discrepancy of serum potassium on mortality has not been evaluated in prior studies. Moreover, stability of potassium levels, rather than those absolute values of baseline, may be more closely relevant to mortality [10]. To the best of our knowledge, there is a lack of study that determines the serum potassium variability with respect to mortality in PD patients.

In this study, we assessed the associations of baseline serum potassium levels with both short- and long-term mortality and evaluated the relationship between serum potassium variability and mortality in incident PD patients.

## Methods

### Ethics Statement

This study was approved by the First Affiliated Hospital of Sun Yet-sen University Institutional Review Boards. All participants provided their written informed consent before inclusion.

### Patients

A total of 1149 incident CAPD patients with 18 years or older and being treated with PD more than 3 months at our centre from January 1, 2006 to December 31, 2010 were studied. All patients were treated with Dianeal solution which does not contain potassium (Baxter China Ltd., Guangzhou, China). We excluded 67 patients because of lack of available baseline potassium and 196 patients with PD-related peritonitis or other acute infection, severe gastrointestinal disease, prescription of diuretics, or concomitant potassium supplementation during the month before the study. 886 patients were enrolled in the final analysis.

Patients were categorized according to baseline serum potassium levels (<3.0, 3.0 to <3.5, 3.5 to <4.0, 4.0 to <4.5, 4.5 to <5.0, and ≥5.0 mEq/L) to examine the association between serum potassium and mortality. Because relatively few patients (17, 1.9%) presented hyperkalemia (serum potassium ≥5.5 mEq/L), we grouped patients with serum potassium ≥5.0 mEq/L into one category for analysis.

To investigate the relation of serum potassium variability and mortality, patients surviving more than one year with at least three check-ups for serum potassium were selected. We excluded 96 patients who commenced PD for less than one year due to following reasons: death (n = 53), transfer to HD (n = 10), renal transplantation (n = 32), and loss to follow-up (n = 1). We also excluded another 99 patients who had less than three times measurement of serum potassium levels because they lived far away from our PD center. Thus, 691 of 886 patients were included in this analysis. Serum potassium variability was expressed as the within-patient standard deviation (SDSP) and the coefficient of variation of serum potassium (CVSP). CVSP was calculated as the ratio of SDSP and the within-patient mean of serum potassium. Patients were split into four categories according quartiles (Q) of CVSP: Q1: <7.5%; Q2∶7.5 to <12.0%; Q3∶12.0 to <16.7%; Q4: ≥16.7%.

### Demographic and Clinical Data

All data were obtained at the time of PD initiation, including demographic details, etiology of end-stage renal disease (ESRD), presence of diabetes, and comorbidity score. Comorbidity was measured using the modified Charlson Comorbidity Index (CCI) [11].

Baseline biochemical data, a standard peritoneal equilibration test (PET) and Kt/V was evaluated in the first 3 months of PD therapy, as previous described [12]. Urine and ultrafiltration volumes during Kt/V test [13], peritoneal transport status (D/P Cr), and adequacy of dialysis [total Kt/V_urea_ and total weekly creatinine clearance (WCCr)] were measured using PD Adequest software (Baxter Healthcare Corporation, Chicago, 1L, USA). Residual glomerular filtration rate (rGFR) was calculated as the average of 24-hour urinary urea and creatinine clearance [14]. Daily exchange volume of peritoneal dialysate was defined as peritoneal dialysis volume per unit of body surface area (PDV/BSA). The number, volume and concentration of glucose exchanges were recorded based on the PD regimen during the first 3 months of PD treatment, and the estimated peritoneal glucose exposure was calculated by the product of the volume and the glucose concentration of each exchange [15]. All biochemical and hematological tests were measured in the biochemical laboratory of the First Affiliated Hospital of Sun Yat-sen University.

### Detection and Management of Hypokalemia and Hyperkalemia

All patients were follow-up every 3 months with serum potassium measured at each visit, and additional measurement would be determined according to patients’ condition. Hypokalemia (serum potassium <3.5 mEq/L) or hyperkalemia was informed to the clinics urgently for immediate recheck or treatment. In brief, patients with a potassium level of 3.0–3.5 mEq/L were commonly prescribed oral potassium supplementation and educated on a high potassium diet. Intravenous potassium was required in patients with a potassium level of <2.5 mEq/L. To avoid bacterial contamination of the dialysis solution, we did not add potassium chloride to solution. For patients with a potassium level ≥5.5 mEq/L, high potassium diet and hyperkalemia generating drugs (angiotensin converting enzyme inhibitors, angiotension receptor antagonists, and potassium-sparing diuretics) would be stopped immediately. Furosemide was prescribed to the patients with urine output >500 ml/day. Intermittent peritoneal dialysis (IPD) or HD was used remove extra potassium in anuric patients or patients with potassium of >6.0 mEq/L. Serum potassium levels were then monitored closely and maintained in the normal range (serum potassium concentration of 3.5 to <5.5 mEq/L).

### Outcomes

Our primary outcomes were all-cause mortality and cardiovascular mortality. Cardiovascular mortality was defined as death from acute heart failure, myocardial infarction, fatal arrhythmia, stroke, peripheral artery disease [16], [17], or sudden death. Survival was defined as the time from enrollment to death or administrative censoring (i.e. transfer to hemodialysis, renal transplantation, transfer to other dialysis centers, loss to follow-up, or end of the study period) at December 31, 2012.

### Statistical Analyses

Results are expressed as mean ± standard deviation (SD) unless otherwise specified. Comparisons between parameters were performed using ANOVA, Kruskal-Wallis H test, or Chi-Square test, as appropriate. The bivariate correlation analysis was tested to assess the associations of demographic and biochemical characteristics with baseline levels of serum potassium. Spearman’s correlation coefficients were used for non-normally distributed variables and Pearson’s correlation coefficients for normally distributed variables. Significant parameters were selected for further multivariable linear regression analysis.

Survival analyses were performed to assess associations of serum potassium levels with all-cause mortality and cardiovascular mortality, with the category of serum potassium 4.0 to <4.5 mEq/L as the reference group. The following models were performed sequentially to estimate mortality rates (per 100 patient-years, pys) during follow-up period according to baseline serum potassium categories: (1) unadjusted; (2) the Cox models were constructed by adjusted for age, gender, body mass index (BMI), diabetic status, CCI, hemoglobin, serum albumin level, high-sensitivity C-reactive protein (hs-CRP), and PDV/BSA, because these parameters were related to serum potassium levels and/or significantly associated with mortality in univariate Cox proportional hazard regression analyses. Conditional Cox regression analysis [18] was carried out to determine the time-stratified effects of baseline serum potassium on mortality. Short-term survival was defined as 1-year patient survival and long-term survival as initiating PD therapy more than one year. In this analysis, the hazard ratios (HRs) for mortality during the first year of dialysis were compared to those during the year after, conditional upon having survived the first year. In addition, to avoid the problems associated with arbitrary selection of categories, the baseline serum potassium was modeled as a continuous predictor by fractional polynomial function to examine its relationship with outcomes [19], [20]. The prognostic significance of serum potassium variability was determined by means of Kaplan-Meier survival curve and Cox proportional hazards regression model. Survival analysis was modeled considering the behavior of serum potassium variability during the first year of follow-up as a predictor of subsequent mortality. All statistical analyses were performed using the SPSS software, version 13.0 (SPSS Inc., Chicago, IL, USA). A P-value <0.05 was considered statistically significant.

## Results

### Baseline Characteristics of Study Cohort

We included 886 incident PD patients with a mean age of 48.5±15.4 years, 42.9% female. Table 1 showed the baseline characteristics of patients stratified by serum potassium levels. 248 patients (27.9%) suffered from hypokalemia, 17 patients (1.9%) presented hyperkalemia (data not shown). Patients with lower potassium were more likely to be older, with more comorbidity, had lower BMI and serum albumin levels, and were treated with higher amount of daily exchange volume and glucose exposure, when compared to patients with normal serum potassium. When patients were further stratified into three groups according to daily urine output at baseline (>500 ml, 100–500 ml, and <100 ml), we found that mean urine output was 673±423 ml/day with 63.2% of the patients having a urine output >500 ml/day and 7.3% of anuric (<100 ml/day) patients. However, neither potassium levels nor hypokalemia prevalence at baseline, and potassium variability was statically different across the groups (data not shown).

**Table 1:** Baseline characteristics of PD patients according to categories of serum potassium.

Variables	Baseline levels of serum potassium (mEq/L)						P-value
	<3.0	3.0 to <3.5	3.5 to <4.0	4.0 to <4.5	4.5 to <5.0	≥5.0	
No. of patients (%)	(n = 66, 7.4%)	(n = 182, 20.5%)	(n = 284, 32.1%)	(n = 220, 24.8%)	(n = 96, 10.8%)	(n = 38, 4.3%)	–
Age (years)	54.1±17.3	50.7±16.5	47.7±15.0	47.1±14.7	47.0±14.5	46.0±12.7	0.005*
Female (%)	35 (53.0%)	85 (46.7%)	120 (42.3%)	92 (41.8%)	35 (36.5%)	13 (34.2%)	0.34
BMI (kg/m^2^)	20.2±2.9	21.4±2.7	21.7±3.2	22.2±3.1	22.6±3.5	22.7±3.4	<0.001*
Etiology of ESRD							0.1
CGN (%)	32 (48.5%)	88 (48.4%)	164 (57.7%)	128 (58.7%)	51 (53.1%)	19 (50.0%)	
DN (%)	15 (22.7%)	46 (25.3%)	50 (17.6%)	52 (23.9%)	25 (26.0%)	11 (28.9%)	
HN (%)	9 (13.6%)	11 (6.0%)	28 (9.9%)	12 (5.5%)	4 (4.2%)	0 (0%)	
Others (%)	10 (15.2%)	37 (20.3%)	42 (14.8%)	26 (11.9%)	16 (16.7%)	8 (21.1%)	
Diabetes (%)	17 (25.8%)	47 (25.8%)	56 (19.7%)	55 (25.0%)	25 (26.0%)	12 (31.6%)	0.38
Charlson Comorbidity Index	4.26±1.87	3.95±2.02	3.49±1.73	3.54±1.88	3.50±2.08	3.32±1.54	0.006*
Use of ACEI/ARB at study initiation (%)	43 (65.2%)	120 (65.9%)	188 (66.2%)	134 (60.9%)	68 (70.8%)	26 (68.4%)	0.35
SBP (mmHg)	142.1±19.8	140.8±15.9	141.2±17.8	140.7±17.3	140.7±15.9	141.2±18.8	0.99
DBP (mmHg)	84.2±14.4	85.4±12.0	84.7±12.9	85.1±13.4	86.1±12.4	86.5±10.7	0.89
Potassium (mEq/L)	2.66±0.25	3.25±0.13	3.69±0.13	4.20±0.15	4.72±0.15	5.51±0.35	<0.001*
Hemoglobin (g/dL)	10.9±2.1	10.8±2.0	11.0±1.9	11.2±2.1	11.3±2.0	10.6±2.0	0. 1
Albumin (g/dL)	3.63±0.53	3.69±0.54	3.80±0.49	3.87±0.43	3.91±0.41	3.85±0.52	<0.001*
Hs-CRP (mg/L)	2.32 (0.55, 8.00)	1.97 (0.66, 8.36)	2.04 (0.63, 6.83)	1.69 (0.58, 6.21)	1.51 (0.52, 4.55)	1.16 (0.49, 5.14)	0.39
FBG (mg/dL)	97.1±37.0	104.8±49.0	95.7±37.3	98.2±41.8	97.1±35.0	81.2±22.0	0.13
rGFR (ml/min/1.73 m^2^)	3.01 (1.08, 4.29)	2.26 (1.13, 3.92)	2.62 (0.99, 4.68)	2.37 (1.13, 3.62)	2.58 (1.27, 4.17)	3.13 (0.78, 4.76)	0.84
Total Kt/V_urea_	2.23±0.61	2.40±0.68	2.29±0.59	2.27±0.62	2.20±0.55	2.50±0.91	0.3
WCCr (L/w/1.73 m^2^)	83.6±29.4	85.4±33.0	83.0±25.9	80.5±25.3	84.8±32.7	96.8±34.7	0.15
Net UF (ml/day)	420 (188, 630)	445 (220, 660)	420 (200, 678)	470 (220, 685)	460 (145, 675)	565 (328, 775)	0.52
Urine output (ml/day)	600 (373, 1000)	630 (400, 803)	620 (400, 878)	620 (370, 870)	690 (390, 978)	500 (230, 880)	0.56
D/P Cr	0.73±0.13	0.70±0.12	0.71±0.11	0.70±0.11	0.71±0.12	0.72±0.11	0.8
PDV/BSA (L/m^2^/day)	4.77±0.64	4.73±0.68	4.71±0.52	4.55±0.63	4.46±0.72	4.49±0.75	<0.001*
Estimated peritonealglucose exposure (g/day)	130.2±27.5	132.2±25.2	133.2±23.1	128.4±24.8	130.0±26.7	131.7±34.2	0.01*

Values expressed as mean ± SD, number (percent), or median (interquartile range). Conversion factors for units: albumin and hemoglobin in g/dL to g/L, × 10; FBG in mg/dL to mmol/L, × 0.05551. No conversion necessary for serum potassium in mEq/L and mmol/L. Abbreviations and definitions: BMI, body mass index; ESRD, end-stage renal disease; CGN, chronic glomerulonephritis; DN, diabetic nephropathy; HN, hypertensive nephrosclerosis; ACEI, angiotensin-converting enzyme inhibitors; ARB, angiotensin II receptor blockades; SBP, systolic blood pressure; DBP, diastolic blood pressure; hs-CRP, high-sensitivity C-reactive protein; FBG, fast blood glucose; rGFR, residual glomerular filtration rate; WCCr, total weekly creatinine clearance; Net UF, net ultrafiltration; D/P Cr, dialysate-to-plasma ratio of creatinine; PDV/BSA, peritoneal dialysis volume per unit of body surface area.

Univariate analysis revealed that baseline serum potassium level was positively correlated with BMI and serum albumin and negatively correlated with age, comorbidity score, hs-CRP, fast blood glucose, and PDV/BSA (Table 2). On multivariate analysis, serum potassium level remained positively associated with BMI (*β* = 0.158; P<0.001) and serum albumin (*β* = 0.117; P = 0.001) and negatively associated with PDV/BSA (*β* = −0.091; P = 0.011) (Table 3).

**Table 2:** Variables correlation with baseline levels of serum potassium.

Variables	*r*	P-value
Age	−0.11	0.001*
Gender	−0.08	0.02*
BMI	0.18	<0.001*
Charlson Comorbidity Index	−0.1	0.004*
Diabetes	−0.01	0.74
Use of ACEI/ARB	−0.02	0.58
SBP (mmHg)	−0.02	0.56
DBP (mmHg)	0.03	0.36
Hemoglobin (g/dL)	0.04	0.24
Albumin (g/dL)	0.16	<0.001*
Hs-CRP (mg/L)	−0.08	0.02*
FBG (mg/dL)	−0.07	0.03*
rGFR (ml/min/1.73 m^2^)	0.02	0.51
Total Kt/V_urea_	−0.02	0.73
WCCr (L/w/1.73 m^2^)	0.02	0.61
Net UF (ml/day)	−0.01	0.8
Urine output (ml/day)	0.05	0.17
D/P Cr	0.01	0.73
PDV/BSA (L/m^2^/day)	−0.15	<0.001*
Estimated peritoneal glucoseexposure (g/day)	−0.02	0.63

Abbreviations and definitions as listed in Table 1.

**Table 3:** Multivariate analysis of clinical measures associated with levels of serum potassium.

Variables	Standard Error	Standardized *β*	*t*	P-value
Age (years)	0.002	−0.084	−1.607	0.108
Gender	0.046	−0.008	−0.221	0.825
BMI (kg/m^2^)	0.007	0.158	4.453	<0.001*
Charlson Comorbidity Index	0.019	0.033	0.608	0.543
Albumin (g/dL)	0.005	0.117	3.377	0.001*
Hs-CRP (mg/L)	0.005	−0.061	−1.808	0.071
FBG (mg/dL)	0.001	−0.05	−1.343	0.18
PDV/BSA(L/m^2^/day)	0.038	−0.091	−2.536	0.011*

Abbreviations and definitions as listed in Table 1.

### Serum Potassium Levels and Mortality

**Figure 1:**
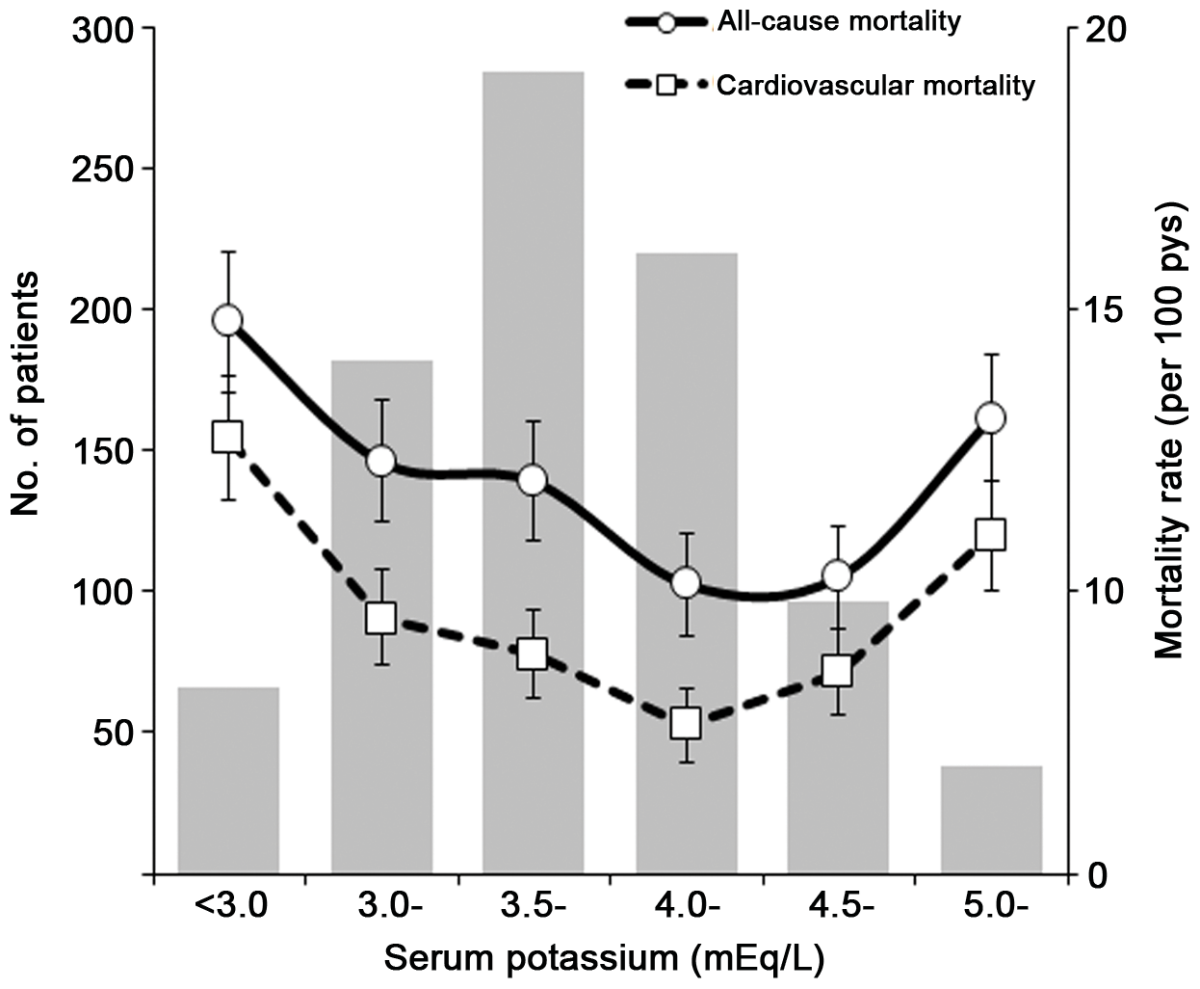
Distribution of baseline serum potassium levels and corresponding mortality rates. Gender- and age- standardized all-cause and cardiovascular mortality rates (per 100 patient-years) with 95% confidence intervals according to serum potassium categories at baseline.

During a median follow-up of 31 months (range: 0.5–81.0 months), 168 patients (19.0%) died. 101 (60.1%) of which were attributed to cardiovascular causes: 72 deaths were cardiac, 27 deaths were due to cerebrovascular disease, and 2 deaths were due to peripheral vascular accident (including one patient with a pulmonary embolism and another with a ruptured abdominal aortic aneurysm). Of all deaths, 53 died within one year, including 32 cardiovascular and 21 non-cardiovascular deaths. As shown in Figure 1 and Table 4, the relationship between baseline serum potassium and all-cause and cardiovascular mortality was U-shaped, with lowest mortality rates observed in patients with potassium levels of 4.0 to 4.5 mEq/L (5.12 and 3.88 death/100 pys, respectively) and highest in that of potassium <3.0 mEq/L. (9.77 and 7.71 death/100 pys, respectively). Both all-cause and cardiovascular mortality rates were higher for potassium levels of ≥5.0 mEq/L (8.06 and 6.0 death/100 pys, respectively), compared with those with levels between 4.0 and 4.5 mEq/L. In unadjusted model, serum potassium levels <3.0 and 3.0 to <3.5 mEq/L were associated with increased risk of all-cause and cardiovascular mortality. After adjustments, the risk of all-cause and cardiovascular death was only significant in those with potassium of <3.0 mEq/L [HR, 1.79 (1.02–3.14) and 2.35 (1.16–4.75), respectively]. In contrast, patients with potassium levels of ≥5.0 mEq/L showed an increased tendency of mortality, although not statistically significant.

**Table 4:** Rates and hazard ratios for all-cause and cardiovascular mortality according to categories of serum potassium.

	Baseline levels of serum potassium (mEq/L)					
	<3.0	3.0 to <3.5	3.5 to <4.0	4.0 to <4.5	4.5 to <5.0	≥5.0
No. of patients	66	182	284	220	96	38
All-cause mortality						
No. of deaths	25	43	49	30	15	6
^ a^Rate (per 100 pys)	9.77 (8.52–11.01)	7.29 (6.21–8.36)	6.94 (5.89–7.99)	5.12 (4.22–6.03)	5.25 (4.34–6.16)	8.06 (6.95–9.19)
Unadjusted HR	2.51 (1.48–4.28)	1.70 (1.06–2.70)	1.34 (0.85–2.11)	1.0 (Reference)	1.16 (0.62–2.15)	1.17 (0.49–2.82)
^ b^Adjusted HR	1.79 (1.02–3.14)	1.15 (0.72–1.86)	1.31 (0.82–2.08)	1.0 (Reference)	1.33 (0.71–2.48)	1.28 (0.53–3.10)
Cardiovascular mortality						
No. of deaths	19	25	26	16	10	5
^ a^Rate (per 100 pys)	7.71 (6.60–8.81)	4.52 (3.67–5.36)	2.63 (1.99–3.28)	3.88 (3.09–4.66)	3.57 (2.82–4.32)	6.0 (5.04–6.98)
Unadjusted HR	3.61 (1.85–7.02)	1.87 (1.00–3.50)	1.34 (0.72–2.49)	1.0 (Reference)	1.47 (0.67–3.25)	1.83 (0.67–5.01)
^ b^Adjusted HR	2.35 (1.16–4.75)	1.25 (0.66–2.37)	1.24 (0.65–2.33)	1.0 (Reference)	1.71 (0.77–3.79)	1.85 (0.67–5.11)

Abbreviations: pys, patient-years; HR, hazard ratio, and other abbreviations and definitions as listed in Table 1.

a Age- and gender-standardized Mortality rate.

b Adjusted for age, gender, BMI, diabetic status, CCI, hemoglobin, serum albumin, hs-CRP, and PDV/BSA.

In conditional Cox analysis, compared with the reference group (4.0 to <4.5 mEq/L), patients with potassium levels of 3.0 to <3.5 mEq/L had a greater than twice-fold increased risk of all-cause death within the first year of follow-up (adjusted HR, 2.80; 95% CI, 1.03–7.65), and much higher at that of <3.0 mEq/L (adjusted HR, 4.34; 95% CI, 1.44–13.1). Moreover, the corresponding adjusted HRs for cardiovascular mortality were 4.61 (95% CI, 1.01–20.9) and 7.17 (95% CI, 1.44–35.8) for serum potassium levels of 3.0–3.5 mEq/L and <3.0 mEq/L, respectively. The increased risk of lower potassium with mortality was evident only during the first year of follow-up. And this relationship was no longer pronounced in the subsequent years of observation (Table 5), indicating the predictive value of hypokalemia for short-term mortality among incident PD patients.

**Table 5:** Association of serum potassium levels and all-cause and cardiovascular mortality by follow-up time.

	Baseline levels of serum potassium (mEq/L)					
	<3.0	3.0 to <3.5	3.5 to <4.0	4.0 to <4.5	4.5 to <5.0	≥5.0
All-cause mortality						
No. of deaths (≤1 years)	10	18	15	5	4	1
^ a^Adjusted HR	4.34 (1.44–13.1)	2.80 (1.03–7.65)	2.10 (0.75–5.88)	1.0 (Reference)	1.81 (0.48–6.89)	1.16 (0.14–10.1)
No. of deaths (>1 years)	15	25	34	25	11	5
^ a^Adjusted HR	1.31 (0.66–2.60)	0.81 (0.46–1.44)	1.16 (0.69–1.97)	1.0 (Reference)	1.24 (0.60–2.55)	1.38(0.52–3.67)
Cardiovascular mortality						
No. of deaths (≤1 years)	7	12	9	2	2	0
^ a^Adjusted HR	7.17 (1.44–35.8)	4.61 (1.01–20.9)	3.12 (0.66–14.7)	1.0 (Reference)	2.45 (0.34–17.7)	–b
No. of deaths (>1 years)	12	13	17	14	8	5
^ a^Adjusted HR	1.70 (0.74–3.92)	0.76 (0.35–1.64)	0.97 (0.47–2.01)	1.0 (Reference)	1.53 (0.63–3.74)	2.29 (0.80–6.53)

Abbreviations: HR, hazard ratio, and other abbreviations and definitions as listed in Table 1.

a Adjusted for age, gender, BMI, diabetic status, CCI, hemoglobin, serum albumin, hs-CRP, and PDV/BSA.

b Number of events in this category is too low to obtain an effect estimate.

**Figure 2:**
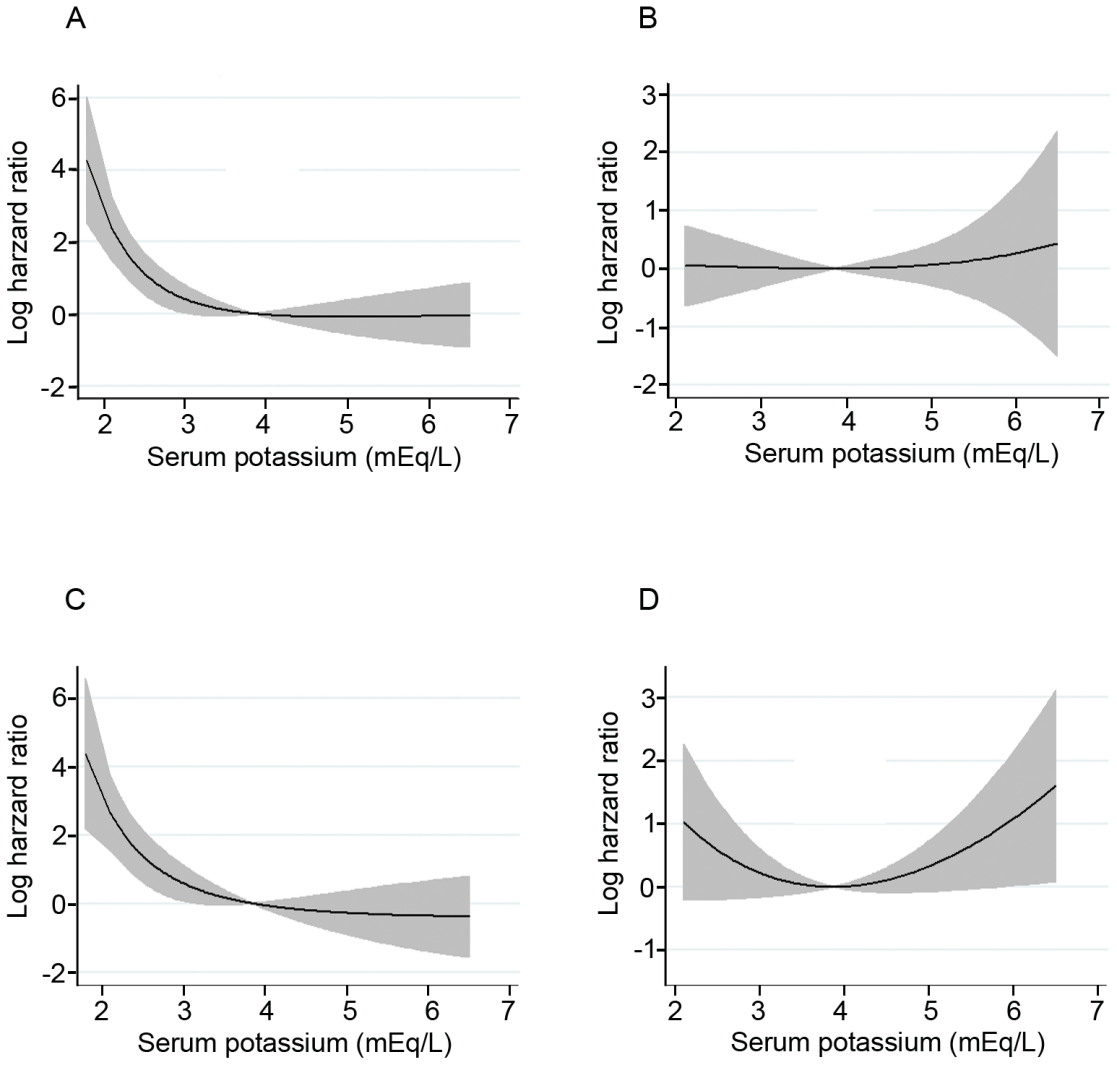
Fractional polynomial graphs depicting the relationship between serum potassium and mortality risk. The relation between serum potassium and all-cause (A, B) and cardiovascular mortality (C, D) in term of short- and long-term mortality rates was expressed by the log-HR ratio. Baseline serum potassium levels were modeled as a continuous variable, and the model was adjusted for age, gender, BMI, diabetic status, CCI, hemoglobin, serum albumin, hs-CRP, and PDV/BSA. Shaded areas indicate the 95% confidence interval.

When baseline serum potassium was modeled as a continuous predictor, as shown in Figure 2, both the short-term all-cause (A) and cardiovascular mortality (C) risk was lowest for serum potassium values of approximately 4 mEq/L and increased steadily with lower serum potassium. However, association of baseline serum potassium levels and long-term mortality became less pronounced (B, D).

### Serum Potassium Variability and Mortality

We further investigated the risk of all-cause mortality according to the quartiles of serum potassium variability (expressed as CVSP). When comparing baseline clinical characteristics between included and excluded patients (99 patients with fewer than 3 serum potassium levels measurements plus 96 patients with less than one year on PD therapy), we found that excluded patients had lower BMI, hemoglobin, serum albumin and serum potassium, while no significant difference between two groups with respect to other variables. As shown in Table 6, the baseline characteristics of included patients across quartiles of CVSP were highly comparable with the exception of age, BMI, comorbidity score, and serum albumin. Patients in the highest quartile of CVSP (≥16.7%) were more likely to be older and had more comorbidity and lower BMI and serum albumin levels (P<0.05 for all).

**Table 6:** Patient baseline characteristics by serum potassium variability during the first year of PD treatment.

Variables	Categories of serum potassium variability (CVSP)				P-value
	Q1 (n = 170)	Q2 (n = 176)	Q3 (n = 173)	Q4 (n = 172)	
Within-patient mean of serum potassium (mEq/L)	3.93±0.49	3.93±0.59	3.93±0.57	3.87±0.61	0.62
SDSP (mEq/L)	0.19±0.07	0.38±0.08	0.55±0.09	0.85±0.25	<0.001*
CVSP (%)	4.94±1.75	9.62±1.30	14.1±1.3	22.1±5.8	<0.001*
Age (years)	45.9±13.3	46.9±15.6	48.1±15.3	52.3±15.8	<0.001*
Female (%)	67 (39.4%)	77 (43.8%)	76 (43.9%)	86 (50.0%)	0.27
BMI (kg/m^2^)	22.1±2.9	22.1±3.3	22.3±3.5	21.3±3.1	0.02*
Etiology of ESRD					0.55
CGN (%)	98 (57.6%)	94 (53.4%)	93 (53.8%)	81 (47.1%)	
DN (%)	34 (20.0%)	35 (19.9%)	39 (22.5%)	50 (29.1%)	
HN (%)	14 (8.2%)	16 (9.1%)	12 (6.9%)	11 (6.4%)	
Others (%)	24 (14.1%)	31 (17.6%)	29 (16.8%)	30 (17.4%)	
Diabetes (%)	36 (21.2%)	39 (22.2%)	41 (23.7%)	53 (30.8%)	0.15
Charlson Comorbidity Index	3.34±1.72	3.55±1.83	3.55±1.79	4.19±2.10	<0.001*
Use of ACEI/ARB at study initiation (%)	108 (63.5%)	119 (67.6%)	114 (65.9%)	110 (64.0%)	0.85
SBP (mmHg)	140.9±17.3	142.0±18.0	138.6±15.3	142.0±17.4	0.21
DBP (mmHg)	85.9±12.4	85.2±14.6	85.2±10.6	85.7±12.2	0.93
Hemoglobin (g/dL)	11.1±1.9	11.2±1.8	11.2±1.9	11.2±2.0	0.83
Albumin (g/dL)	3.91±0.46	3.85±0.43	3.86±0.45	3.75±0.46	0.009*
Hs-CRP (mg/L)	1.64 (0.64, 6.52)	2.04 (0.69, 6.46)	2.45 (0.52, 8.22)	1.93 (0.61, 6.20)	0.74
FBG (mg/dL)	95.5±35.8	95.6±37.8	96.7±42.0	104.3±45.8	0.14
rGFR (ml/min/1.73 m^2^)	2.36 (0.98, 4.41)	2.09 (0.94, 3.68)	2.64 (1.13, 4.37)	2.58 (1.19, 4.54)	0.29
Total Kt/V_urea_	2.32±0.72	2.27±0.60	2.22±0.54	2.26±0.60	0.73
WCCr (L/w/1.73 m^2^)	81.5±27.9	81.4±24.6	82.8±29.7	86.4±31.4	0.53
Net UF (ml/day)	400 (200, 650)	450 (200, 648)	460 (165, 670)	570 (210, 750)	0.16
Urine output (ml/day)	670 (378, 900)	605 (393, 930)	620 (385, 880)	580 (323, 795)	0.36
D/P Cr	0.70±0.12	0.72±0.12	0.71±0.11	0.71±0.11	0.55
PDV/BSA (L/m^2^/day)	4.62±0.57	4.63±0.57	4.58±0.63	4.71±0.70	0.27
Estimated peritoneal glucose exposure (g/day)	131.2±24.2	131.7±25.7	130.9±23.3	131.7±29.4	0.98

Note: Values expressed as mean ± SD, number (percent), or median (interquartile range). Conversion factors for units: albumin and hemoglobin in g/dL to g/L, × 10; FBG in mg/dL to mmol/L, × 0.05551. No conversion necessary for serum potassium in mEq/L and mmol/L.

Abbreviations: CVSP, coefficient variation of serum potassium; SDSP, standard deviation of serum potassium; and other abbreviations as listed in Table 1.

**Figure 3:**
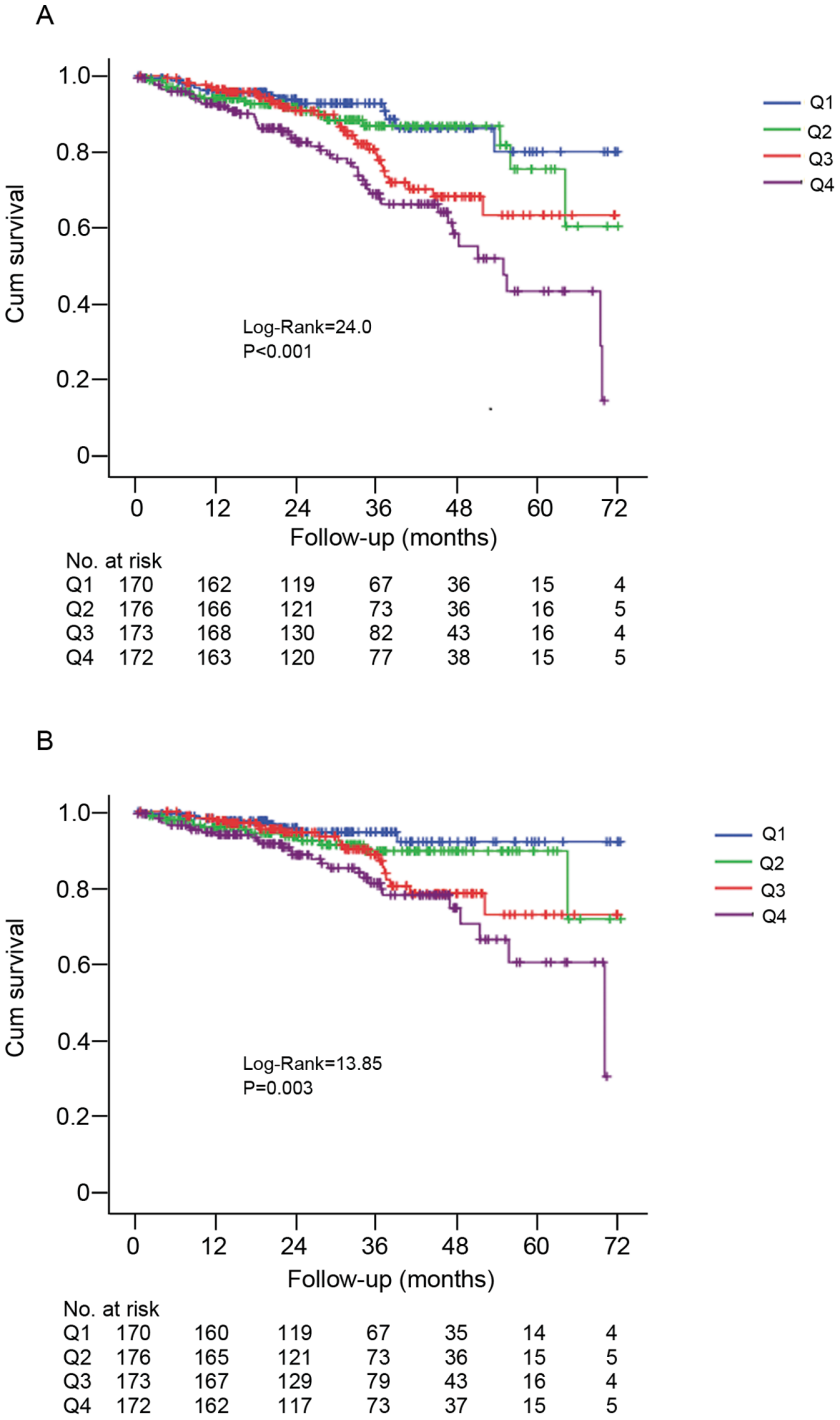
Kaplan-Meier survival curves for mortality according to serum potassium variability. (A) all-cause mortality; (B) cardiovascular mortality. Patients were split into four categories according quartiles (Q) of coefficient of variation of serum potassium (CVSP): Q1: <7.5%; Q2∶7.5 to <12.0%; Q3∶12.0 to <16.7%; Q4: ≥16.7%. The P values refer to the significance of the log-rank test across quartiles.

Figure 3 displayed the Kaplan–Meier survival curves for all-cause (A) and cardiovascular (B) mortality across quartiles of CVSP (Q1, Q2, Q3, and Q4), indicating that patients with higher serum potassium variability (Q3 and Q4) had significantly poorer all-cause (P<0.001) and cardiovascular mortality (P = 0.003), compared to those with relatively stable potassium levels (Q1).

**Figure 4:**
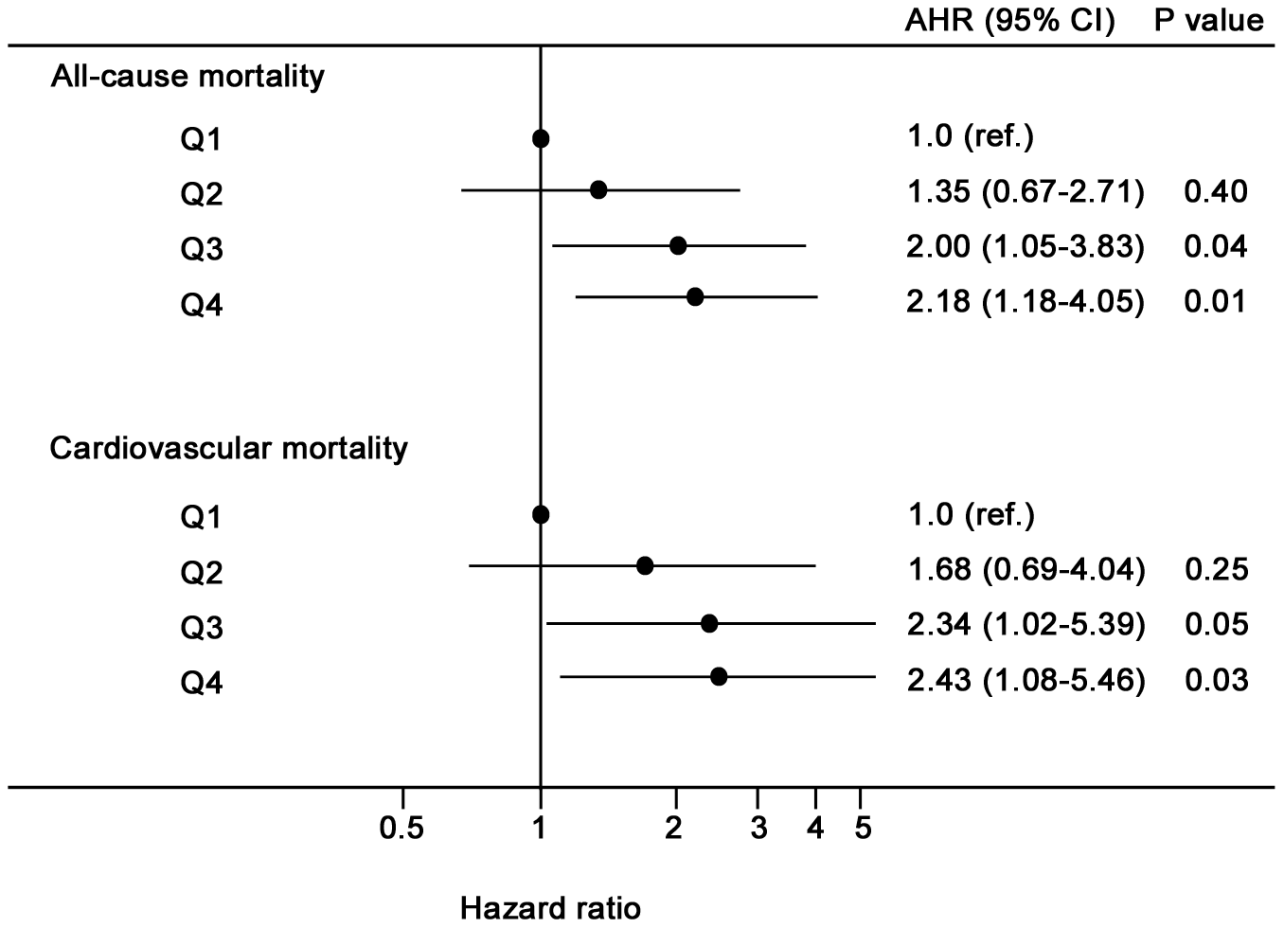
Hazard ratio for all-cause and cardiovascular mortality according to serum potassium variability. Adjustments included age at initiation of PD, gender, BMI, diabetes status, CCI, hemoglobin, serum albumin, hs-CRP, PDV/BSA, and within-patient mean of serum potassium. Q1: (CVSP) <7.5%; Q2: (CVSP) 7.5 to <12.0%; Q3: (CVSP) 12.0 to <16.7%; Q4: (CVSP) ≥16.7%.

The relative risks of mortality in relation to serum potassium variability showed in Figure 4, with the Q1 group as a reference. All-cause mortality risk in the Q3 group was higher and appeared to increase steadily in the Q4 group, with adjusted hazard ratios of 2.00 (1.05–3.83) and 2.18 (1.18–4.05), respectively, independently of the within-patient mean of serum potassium. However, the Q2 group was not associated with a higher risk for all-cause death. Similar results were observed with regard to cardiovascular mortality, with adjusted hazard ratios 2.34 (1.02–5.39) and 2.43 (1.08–5.46) for the Q3 and Q4 groups, respectively.

## Discussion

In the present study, we demonstrated that 27.9% of incident PD patients presented hypokalemia. There was a U-shaped association of baseline serum potassium levels and all-cause and cardiovascular mortality, with the highest mortality rate observed for potassium levels <3.0 mEq/L. After adjustment for confounders, the relationship between hypokalemia and mortality risk was evident only during the first year, but not thereafter. Furthermore, higher serum potassium variability conferred an increased mortality risk, which was independent of the average serum potassium levels.

The risk factors for hypokalemia in PD patients are complex, many factors are involved. Associations of chronic inflammation and malnutrition with potassium levels have been described previously [8], [21], [22], [23]. Indeed, we found that patients with a lower potassium levels were older and had more comorbidities, lower albumin and BMI. Given that peritoneal dialysate is potassium-free, ongoing losses of potassium into dialysate may contribute to hypokalemia in some patients. Our results were in accordance with previous studies [24], [25], revealing that an elevated dialysis dose was independently related to decreased serum potassium levels. In addition, potassium redistribution into the intracellular compartment, stimulated by insulin release due to continuous peritoneal glucose infusion was thought to be another important risk factor for hypokalemia [23]. However, we were unable to demonstrate a relationship between serum potassium and daily glucose exposure. Because we did not measure the absolute amount of peritoneal glucose absorption, and the effects of cellular uptake on potassium levels couldn’t be excluded. Moreover, in line with previous studies [8], [26], we did not found any association between serum potassium levels and urine volume or residual renal function, suggesting that the removal of potassium from urinary might be insufficient to develop hypokalemia in our study population.

A relation of serum potassium levels with the risk of death has been reported by Szeto *et al.* [8], indicating a higher risk for all-cause mortality in PD patients with baseline serum potassium <3.5 mEq/L. In the most recent and robust evidence from a large, contemporary cohort of dialysis population showed a clear U-shaped relationship between time-averaged serum potassium levels and all-cause and cardiovascular mortality in prevalent PD patients, and the lowest mortality was observed in patients with serum potassium of 4.0–4.5 mEq/L, while increased mortality for serum potassium <4.0 mEq/L and ≥5.5 mEq/L [9]. Our results were somewhat consistent with previous study that a U-shaped association of baseline serum potassium and age - and gender- standardized all-cause and cardiovascular mortality rates in incident PD patients, with the highest mortality rate observed for potassium levels <3.0 mEq/L. However, our study showed that patients with potassium levels ≥5.0 mEq/L had an increased tendency of mortality rate, though without statistically significance, probably due to the small number of PD patients in this category (4.3% of the total patients).

Abnormal serum potassium levels most prominently affect the cardiovascular system and have been implicated in many aspects of cardiovascular disease including atrial fibrillation, stroke, heart attack, hypertension, and sudden cardiac death [27]. Therefore, any abnormalities in serum potassium are usually corrected as soon as possible in our clinical practices. To our knowledge, this is the first study to examine the relationship between time discrepancy of serum potassium levels and mortality in PD patients. We found that the association between hypokalemia and mortality risk was evident only within the first year, but vanished in the subsequent years. This relationship remained significant after adjustment for confounders. Our data revealed the importance of early correction of abnormal serum potassium in this population. Consistently, we demonstrated that patients with lower or higher serum potassium levels had increased rates of cardiovascular mortality. Another potential explanation is malnutrition, one of the confounders, termed as a “short-term killer” [28], [29], because hypokalemia was closely associated with malnutrition and severe coexisting comorbid conditions, which might be responsible for the poor short-term outcome.

It has been reported that the variability of heart rate [30], blood pressure [31], hemoglobin [32], as well as variability of serum calcium and phosphorus [33] are closely associated with clinical outcomes. Additionally, highest risk of death was found on the days following the longest interval without dialysis [34]. Rapid reduction of potassium after HD may be one of vital contributors, since the excessive variability in serum potassium levels can enhance the risk for cardiac arrhythmia and sudden death. However, the relationship between distribution of serum potassium variability and mortality risk has not been investigated in PD patients. We found that patients with a relative stable serum potassium levels during one year’s observation appeared to have a pronouncedly better survival. An excessive fluctuation in serum potassium was associated with significantly increased mortality, which was independent of the within-patient average serum potassium levels.

Nonetheless, our study has several limitations. First, an observational study cannot prove causality. Second, there is a potential selection bias when we evaluated the association between serum potassium variability and mortality. Because we enrolled patients who had received PD for more than one year with at least three check-ups of serum potassium, patients with relatively lower or higher serum potassium levels might died within the first year of therapy. Thus, these data may underestimate the negative roles of serum potassium variability in mortality. Third, relatively few patients presented abnormally high serum potassium levels. Hence, a small number of patients with hyperkalemia may result in the lack of significant association with mortality. Finally, factors affecting serum potassium levels, such as dietary intake, loss via dialysate and urine, were not examined in the present study.

In conclusion, our study demonstrates for the first time that association between hypokalemia at baseline and mortality risk is evident only during the first year of follow-up period. In addition, the higher serum potassium variability may contribute substantially to both all-cause and cardiovascular mortality risk in this population. Our findings emphasize the importance of early achieving the optimal and stable serum potassium levels in patients initiating PD therapy.

## References

[1] Korgaonkar S, Tilea A, Gillespie BW, Kiser M, Eisele G, et al (2010) Serum potassium and outcomes in CKD: insights from the RRI-CKD cohort study. Clin J Am Soc Nephrol 5: 762–769. 20203167 10.2215/CJN.05850809 PMC2863985

[2] Kovesdy CP, Regidor DL, Mehrotra R, Jing J, McAllister CJ, et al (2007) Serum and dialysate potassium concentrations and survival in hemodialysis patients. Clin J Am Soc Nephrol 2: 999–1007. 17702709 10.2215/CJN.04451206

[3] Oreopoulos DG, Khanna R, Williams P, Vas SI (1982) Continuous ambulatory peritoneal dialysis - 1981. Nephron 30: 293–303. 7110460 10.1159/000182504

[4] Rostand SG (1983) Profound hypokalemia in continuous ambulatory peritoneal dialysis. Arch Intern Med 143: 377–378. 6824407

[5] Khan AN, Bernardini J, Johnston JR, Piraino B (1996) Hypokalemia in peritoneal dialysis patients. Perit Dial Int 16: 652. 8981546

[6] Go AS, Chertow GM, Fan D, McCulloch CE, Hsu CY (2004) Chronic kidney disease and the risks of death, cardiovascular events, and hospitalization. N Engl J Med 351: 1296–1305. 15385656 10.1056/NEJMoa041031

[7] Harrison TR, Pilcher C, Ewing G (1930) STUDIES IN CONGESTIVE HEART FAILURE: IV. The Potassium Content of Skeletal and Cardiac Muscle. J Clin Invest 8: 325–335. 16693898 10.1172/JCI100267 PMC424623

[8] Szeto CC, Chow KM, Kwan BC, Leung CB, Chung KY, et al (2005) Hypokalemia in Chinese peritoneal dialysis patients: prevalence and prognostic implication. Am J Kidney Dis 46: 128–135. 15983966 10.1053/j.ajkd.2005.03.015

[9] Torlen K, Kalantar-Zadeh K, Molnar MZ, Vashistha T, Mehrotra R (2012) Serum potassium and cause-specific mortality in a large peritoneal dialysis cohort. Clin J Am Soc Nephrol 7: 1272–1284. 22626960 10.2215/CJN.00960112 PMC3408121

[10] Kwan BC, Szeto CC (2012) Dialysis: Hypokalaemia and cardiac risk in peritoneal dialysis patients. Nat Rev Nephrol 8: 501–503. 22801950 10.1038/nrneph.2012.159

[11] Beddhu S, Zeidel ML, Saul M, Seddon P, Samore MH, et al (2002) The effects of comorbid conditions on the outcomes of patients undergoing peritoneal dialysis. Am J Med 112: 696–701. 12079709 10.1016/s0002-9343(02)01097-5

[12] Rocco MV, Jordan JR, Burkart JM (1994) Determination of peritoneal transport characteristics with 24-hour dialysate collections: dialysis adequacy and transport test. J Am Soc Nephrol 5: 1333–1338. 7893998 10.1681/ASN.V561333

[13] Konings CJ, Kooman JP, Schonck M, Struijk DG, Gladziwa U, et al (2003) Fluid status in CAPD patients is related to peritoneal transport and residual renal function: evidence from a longitudinal study. Nephrol Dial Transplant 18: 797–803. 12637651 10.1093/ndt/gfg147

[14] van Olden RW, Krediet RT, Struijk DG, Arisz L (1996) Measurement of residual renal function in patients treated with continuous ambulatory peritoneal dialysis. J Am Soc Nephrol 7: 745–750. 8738810 10.1681/ASN.V75745

[15] Davies SJ, Phillips L, Naish PF, Russell GI (2001) Peritoneal glucose exposure and changes in membrane solute transport with time on peritoneal dialysis. J Am Soc Nephrol 12: 1046–1051. 11316864 10.1681/ASN.V1251046

[16] Wang AY, Wang M, Woo J, Lam CW, Li PK, et al (2003) Cardiac valve calcification as an important predictor for all-cause mortality and cardiovascular mortality in long-term peritoneal dialysis patients: a prospective study. J Am Soc Nephrol 14: 159–168. 12506148 10.1097/01.asn.0000038685.95946.83

[17] Chertow GM, Block GA, Correa-Rotter R, Drueke TB, Floege J, et al (2012) Effect of cinacalcet on cardiovascular disease in patients undergoing dialysis. N Engl J Med 367: 2482–2494. 23121374 10.1056/NEJMoa1205624

[18] Dekker FW, de Mutsert R, van Dijk PC, Zoccali C, Jager KJ (2008) Survival analysis: time-dependent effects and time-varying risk factors. Kidney Int 74: 994–997. 18633346 10.1038/ki.2008.328

[19] McDonald SP, Collins JF, Johnson DW (2003) Obesity is associated with worse peritoneal dialysis outcomes in the Australia and New Zealand patient populations. J Am Soc Nephrol 14: 2894–2901. 14569099 10.1097/01.asn.0000091587.55159.5f

[20] Royston P (2000) A strategy for modelling the effect of a continuous covariate in medicine and epidemiology. Statistics in medicine 19: 1831–1847. 10867674 10.1002/1097-0258(20000730)19:14<1831::aid-sim502>3.0.co;2-1

[21] Chuang YW, Shu KH, Yu TM, Cheng CH, Chen CH (2009) Hypokalaemia: an independent risk factor of Enterobacteriaceae peritonitis in CAPD patients. Nephrol Dial Transplant 24: 1603–1608. 19103738 10.1093/ndt/gfn709

[22] Jung JY, Chang JH, Lee HH, Chung W, Kim S (2009) De novo hypokalemia in incident peritoneal dialysis patients: a 1-year observational study. Electrolyte Blood Press 7: 73–78. 21468189 10.5049/EBP.2009.7.2.73 PMC3041491

[23] Tziviskou E, Musso C, Bellizzi V, Khandelwal M, Wang T, et al (2003) Prevalence and pathogenesis of hypokalemia in patients on chronic peritoneal dialysis: one center's experience and review of the literature. Int Urol Nephrol 35: 429–434. 15160552 10.1023/b:urol.0000022867.93739.03

[24] Gao H, Lew SQ, Bosch JP (1999) Biochemical parameters, nutritional status and efficiency of dialysis in CAPD and CCPD patients. Am J Nephrol 19: 7–12. 10085443 10.1159/000013418

[25] Newman LN, Weiss MF, Berger J, Priester A, Negrea LA, et al (2000) The law of unintended consequences in action: increase in incidence of hypokalemia with improved adequacy of dialysis. Adv Perit Dial 16: 134–137. 11045278

[26] Li PK, Chow KM, Wong TY, Leung CB, Szeto CC (2003) Effects of an angiotensin-converting enzyme inhibitor on residual renal function in patients receiving peritoneal dialysis. A randomized, controlled study. Ann Intern Med 139: 105–112. 12859160 10.7326/0003-4819-139-2-200307150-00010

[27] Sica DA, Struthers AD, Cushman WC, Wood M, Banas JS Jr, et al (2002) Importance of potassium in cardiovascular disease. J Clin Hypertens (Greenwich) 4: 198–206. 12045369 10.1111/j.1524-6175.2002.01728.x PMC8101903

[28] Fleischmann EH, Bower JD, Salahudeen AK (2001) Risk factor paradox in hemodialysis: better nutrition as a partial explanation. ASAIO J 47: 74–81. 11199320 10.1097/00002480-200101000-00016

[29] Kalantar-Zadeh K, Block G, Humphreys MH, Kopple JD (2003) Reverse epidemiology of cardiovascular risk factors in maintenance dialysis patients. Kidney Int 63: 793–808. 12631061 10.1046/j.1523-1755.2003.00803.x

[30] Chan CT (2008) Heart rate variability in patients with end-stage renal disease: an emerging predictive tool for sudden cardiac death? Nephrol Dial Transplant 23: 3061–3062. 18503010 10.1093/ndt/gfn280

[31] Di Iorio B, Pota A, Sirico ML, Torraca S, Di Micco L, et al (2012) Blood pressure variability and outcomes in chronic kidney disease. Nephrol Dial Transplant 27: 4404–4410. 22962409 10.1093/ndt/gfs328

[32] Kainz A, Mayer B, Kramar R, Oberbauer R (2010) Association of ESA hypo-responsiveness and haemoglobin variability with mortality in haemodialysis patients. Nephrol Dial Transplant 25: 3701–3706. 20507852 10.1093/ndt/gfq287 PMC3360143

[33] Kalantar-Zadeh K, Kuwae N, Regidor DL, Kovesdy CP, Kilpatrick RD, et al (2006) Survival predictability of time-varying indicators of bone disease in maintenance hemodialysis patients. Kidney Int 70: 771–780. 16820797 10.1038/sj.ki.5001514

[34] Bleyer AJ, Russell GB, Satko SG (1999) Sudden and cardiac death rates in hemodialysis patients. Kidney Int 55: 1553–1559. 10201022 10.1046/j.1523-1755.1999.00391.x

